# Method Development and Validation for the Simultaneous Analysis of Miconazole Nitrate, Hydrocortisone and Chlorocresol in a Pharmaceutical Topical Cream by Reverse Phase Liquid Chromatography

**DOI:** 10.1002/ansa.70085

**Published:** 2026-04-27

**Authors:** Netai Mukaratirwa‐Muchanyereyi, Pretty Chihiya, Stephen Nyoni

**Affiliations:** ^1^ Department of Chemistry Bindura University of Science Education Bindura Zimbabwe; ^2^ Department of Chemistry Chinhoyi University of Technology Chinhoyi Zimbabwe

**Keywords:** method development, RP liquid chromatography, separation, validation

## Abstract

Miconazole/Hydrocortisone (HCA) cream is a pharmaceutical formulation developed as a combination of three major components (HCA, miconazole nitrate [MCN] and chlorocresol [CHL]). In some instances, reference monographs used for referencing the quantification of pharmaceutical molecules focus on individual analysis of molecules. Therefore, there is need to develop, optimize and validate methods that simultaneously separate constituents of a combined formulation. In this study, a method for the separation of HCA, MCN and CHL in miconazole/HCA cream was developed by investigating the effect of mobile phase, stationary phase, wavelength and gradient program on the separation of HCA, MCN and CHL in miconazole/HCA cream. A 10 mM ammonium acetate (AAC) buffer pH 5.0 was used. Acetonitrile was used as the organic modifier. An injection volume of 10 µL was used on a C18, 15 cm × 4.6 mm Zorbax. The optimum conditions for reverse phase liquid chromatography were obtained as follows: injection volume (10 µL), column temperature (30°C), flow rate (1 mL/min), gradient elution program: 0 min, 60AAC:40ACN; 4 min, 60AAC:40ACN; 9 min, 5AAC:95ACN; 13 min, 5AAC:95ACN using a C18 stationary phase, UV detector, 5 µm stationary phase pore size and post run of 3 min.

## Graphical abstract

Development and optimization of a reverse‐brphase chromatography method is done for the simultaneous analysis of hydrocortisone, miconazole nitrate, chlorocresol in a pharmaceutical topical cream. Key parameters are evaluated in this study. The optimum conditions are; volume (10 µL), temperature (30°C), flow rate (1 ml/min), gradient elution program: (0 min; 60AAC:40ACN), (4 min; 60AAC:40ACN), (9 min; 5AAC:95ACN), (13 min; 5AAC: 95ACN) using a C18 stationary phase, UV detector, 5 µm stationary phase pore size and post run (3 min).

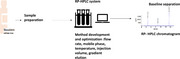

## 1 Introduction

Miconazole/Hydrocortisone (HCA) cream is an antifungal cream consisting of HCA (1%), miconazole nitrate (MCN) (2%) and preserved with chlorocresol (CHL) (0.1%). In most cases, pharmaceutical formulations are developed as combinations of two or more active pharmaceutical ingredients and preservatives. This is done to increase effectiveness of a particular drug. MCN is an antifungal synthetic derivative of imidazole and a salt of miconazole as a nitrate. It is used in the treatment of candida skin infections by selectively affecting the integrity of fungal cell membranes [1, 2, 3]. HCA is a natural corticosteroid with a high potent anti‐inflammatory and anti‐pruriginous action [4, 5]. Miconazole is poorly absorbed after application [6]; HCA penetrates well into the skin [7], hence the need to have a formulation with both molecules as active pharmaceutical ingredients. Miconazole/HCA cream is preserved with CHL. It has been revealed that simultaneous methods to separate MCN and HCA from the cream sample matrix were developed in the past [8, 9, 10, 11, 12, 13, 14, 15, 16], such as reverse phase high‐performance liquid chromatography (RP‐HPLC), high‐performance thin‐layer chromatography (HPTLC), derivative UV–Vis spectrophotometry and absorbance ratio spectrophotometry. However, these methods excluded the separation of CHL. The basics of liquid chromatography method development and optimization in analytical separation sciences have already been described by many researchers [17, 18]. It has been also noted that most reference monographs for referencing the quantification of pharmaceutical molecules focus only on the analysis of individual molecules. A review on method development, validation, optimization and applications of HPLC was reported [19]. The review highlighted the need for method development and the parameters to be evaluated. Therefore, this study intends to develop and validate a method that can simultaneously separate HCA, MCN and CHL. This will allow the reduction of errors and expenses incurred when analysing the molecules individually as well as shorten the analysis time. The method's applicability is confirmed by testing a commercial cream and comparing the results obtained from quantifying the three molecules individually through referencing the United States Pharmacopeia. The Student *t*‐test and F‐test statistical tools were used for comparing the results.

## 2 Materials, Instrumentation and Methods

### 2.1 Materials and Reagents

MCN USP, HCA USP, CHL USP, pharmaceutical formulation (topical cream), methanol HPLC grade, acetonitrile HPLC grade, ammonium acetate (AAC) A.R., acetic acid A.R.

### 2.2 Instrumentation

#### 2.2.1 Method Development and Optimization

The wavelength, column packing, mobile phase, flow rate, injection volume, column temperature and the gradient program were optimized before method validation. HPLC optimization was done on the Agilent 1260 Infinity 11 HPLC manufactured by Agilent Technologies in Germany. Reverse phase chromatography was used; a 15 cm C18 column and C8 column were used to optimize the method. The specific parameters were changed and monitored based on compendia specifications in order to come up with the best method for the separation of the three molecules.

#### 2.2.2 Extraction Solvent and Extraction Method Selection

Methanol was used as the solvent of extraction. The sample was extracted by heating in a water bath after the addition of methanol to disperse the cream.

#### 2.2.3 Wavelength Selection

The absorption maxima of the three molecules were established by scanning the three molecules dissolved in methanol on the UV–Vis spectrophotometer Model Evo300 manufactured by Thermo Fisher Scientific (USA) in the range of 200–400 nm. Methanol was used as the blank for all the three molecules.

#### 2.2.4 Mobile Phase Selection

A buffer and an organic solvent were selected on the basis of the polarity of the molecules. Both methanol and acetonitrile were optimized as organic solvents for the method. Acetonitrile was selected as the organic solvent. A 10 mM acetate buffer and a 10 mM phosphate buffer were optimized for the method. The selection of the buffer pH was based on the p*K*a of the three molecules, that is, CHL 9.2, HCA 12.58 and MCN 6.65. The buffer pH3, pH4.5, pH5, pH7 and pH9 were optimized. The best was chosen for the method.

#### 2.2.5 Flow‐Rate, Injection Volume and Column Temperature Selection

The flow rate of the method was optimized by changing it from 1 to 1.5 mL/min. The injection volume was also changed from 5, 10 and 20 µL. The column temperature was changed from 25°C, 30°C and 35°C. After optimizing the mobile phase, a gradient program was optimized so as to shorten the run time. The optimum conditions from the study were used to validate the method. The extraction of the cream was done using methanol in a water bath at 60°C for 10 min.

### 2.3 Chromatographic Conditions and Instruments

A 10 mM AAC buffer pH 5.0 was used. Acetonitrile was used as the organic modifier. An injection volume of 10 µL was used on a C18, 15 cm × 4.6 mm Zorbax Eclipse Plus column. A UV detector wavelength of 230 nm was used on all the three molecules. The column temperature was set at 35°C. A flow rate of 1 mL/min was used. The gradient program used for analysis was 0 min, 60AAC:40ACN; 4 min, 60AAC:40ACN; 9 min, 5AAC:95ACN; 13 min, 5AAC:95ACN and post‐run time of 3 min.

### 2.4 Method Validation

#### 2.4.1 Specificity

About 2.5 g of the placebo was weighed into a 100 mL volumetric flask and extracted in 65% of the methanol diluent. The extract mixture was sonicated for 30 min, allowed to cool to room temperature and topped up to volume with methanol. The solution was filtered and injected once into the chromatographic system. The diluent (methanol) was injected once into the chromatographic system.

#### 2.4.2 Precision

##### 2.4.2.1 Preparation of Standard Solution

About 50 mg of MCN and 25 mg of HCA reference standards were weighed into a 100 mL volumetric flask. Methanol (65%) was added and sonicated for 30 min to dissolve the mixture. The solution was not made up to volume. In a separate 100 mL volumetric flask, about 25 mg of CHL was weighed, methanol (65%) was added, and the mixture was dissolved by sonication for 30 min and made up to the mark with methanol. About 10 mL of the CHL solution was pipetted into the MCN and HCA volumetric flask, and the solution made up to volume with methanol. The standard solution was injected five times into the chromatographic system.

##### 2.4.2.2 Sample Preparation

About 2.5 g of the cream was weighed into a beaker, and 40 mL of methanol was added. The cream was dispersed by heating in a water bath for 10 min at 60°C. The dispersed cream was transferred into a 100 mL volumetric flask and sonicated for 30 min. The sonicated solution was allowed to cool, made up to the mark and filtered. The filtrate was transferred into a vial and injected into the chromatographic system using the chromatographic conditions provided. Six samples were prepared.

#### 2.4.3 Intermediate Precision

The analysis was done on a different day but using the same conditions.

#### 2.4.4 Linearity and Range

The solutions were prepared covering a range of 80%–120% of the analyte concentration and injected into the chromatographic system. The solutions were prepared in triplicate at each concentration and injected once.

##### 2.4.4.1 Preparation of Standard Stock Solution for Determination of Linearity and Range

About 500 mg of MCN and 250 mg of HCA reference standard were weighed into a 100 mL volumetric flask. A total of 65% of methanol was added and sonicated for 30 min to dissolve. The solution was not made up to volume. In a separate 100 mL volumetric flask, about 250 mg of CHL was weighed, 65% of methanol was added, and the mixture was dissolved by sonication for 30 min and made up to volume with methanol. A volume of 10 mL of the CHL solution was pipetted into the MCN and HCA volumetric flask, and the solution was made up to the mark.

##### 2.4.4.2 Preparation of Solutions for Linearity Testing

The solutions were prepared as shown in Table 1.

**TABLE 1:** Preparation of solutions for linearity testing.

Dilution (methanol diluent)	% Concentration
2 mL (stock) → 25 mL	80
9 mL (stock) → 100 mL	90
2 mL (stock) → 20 mL	100
11 mL (stock) → 110 mL	110
3 mL (stock) → 25 mL	120

#### 2.4.5 Accuracy

##### 2.4.5.1 Preparation of Standard Stock Solution for Determination of Method Accuracy

Concentrations of 80%, 100% and 120% were spiked into the placebo and injected into the system. These were prepared in triplicates and injected once into the chromatographic system. About 500 mg of MCN and 250 mg of HCA reference standard were weighed into a 100 mL volumetric flask; methanol (65%) was added and sonicated for 30 min to dissolve. The solution was not be made up to mark. In a separate 100 mL volumetric flask, about 250 mg of CHL was weighed, methanol (65%) was added, the mixture was dissolved by sonication for 30 min and made up to the mark with methanol. A volume of 10 mL of the CHL solution was pipetted into the MCN and HCA volumetric flask, and the solution was made up to the mark.

##### 2.4.5.2 Preparation of Solutions for Accuracy Testing

The solution preparation procedure is shown in Table 2.

**TABLE 2:** Preparation of solutions for accuracy testing.

Dilution (methanol diluent)	% Concentration
8 mL (stock) + 2.5 g placebo → 100 mL	80
2 mL (stock) + 2.5 g placebo → 20 mL	100
6 mL (stock) + 2.5 g placebo → 50 mL	120

#### 2.4.6 Robustness

The standard and sample solutions were prepared the same way as the ones for precision (Section 2.4.2). The standard solution was injected five times and the sample injected twice at 100% concentration, and method parameters were varied. The parameters, which were varied as per ICH recommendations, are column temperature ±2°C (28°C and 32°C) and the flow rate at ±0.1 mL/min (0.9 and 1.1 mL).

### 2.5 Method Applicability

The three molecules were analysed individually using USP procedures [20] and other methods in literature against the new validated method of analysis. The results from these independent methods were compared using the Student's *t*‐test and *F*‐test. A commercial cream was analysed using the developed method, and the results were compared with those from the existing method.

#### 2.5.1 HCA Method of Analysis in the Cream as per USP

Chromatographic conditions: detector wavelength UV @ 254; column 3.9 mm × 30 cm; injection volume 10 µL; mobile phase: acetonitrile:water (25:75); diluent: dilute methanol (1 in 2).

##### 2.5.1.1 Standard Preparation for Analysis of HCA in the Cream as per USP

About 50 mg of HCA was weighed into a 100 mL volumetric flask, 65% methanol was added and sonicated for 30 min. The solution was made up to volume with methanol. The solution was further diluted by pipetting 10 mL into a 100 mL and diluting with methanol.

##### 2.5.1.2 Sample Preparation for Analysis of HCA in the Cream as per USP

About 1 g of the cream was transferred into a 150 mL beaker. This was mixed with 40 mL of methanol, and the mixture was allowed to disperse by heating on a steam bath. The mixture was cooled to room temperature and filtered into a 100 mL volumetric flask. The extraction was repeated twice with 20 mL portions of methanol and made up to the mark with methanol. The solution was transferred into a vial and analysed using the predetermined chromatographic conditions provided.

#### 2.5.2 MCN Method of Analysis as per USP

Chromatographic conditions: detector UV @ 225; column 4.6 mm × 25 cm C18; column temperature 45°C; flow rate 1 mL/min; injection volume 10 µL; mobile phase: methanol:acetonitrile:tetrahydrofuran:buffer (5:4:3:8); buffer: trimethylamine and water (10:100).

##### 2.5.2.1 Standard Preparation for Analysis of MCN in the Cream as per USP

About 28 mg of MCN reference standard and 2 mg of benzoic acid were weighed into a 100 mL and diluted in mobile phase.

##### 2.5.2.2 Sample Preparation for Analysis of MCN in the Cream as per USP

About 1.4 g of the cream was weighed into a beaker, and 40 mL of the mobile phase was added and dispersed on a steam bath. The solution was transferred into a 100 mL volumetric flask, allowed to cool and made up to the mark. Finally, the solution was filtered into a vial and analysed using the predetermined chromatographic conditions provided.

#### 2.5.3 CHL Method of Analysis

Chromatographic conditions: mobile phase: water:methanol (55:45); flow rate 1 mL/min; injection volume 10 µL; column temperature 30°C; Zorbax C18 column 150 × 4.6 mm^2^; detector wavelength 240 nm.

##### 2.5.3.1 Standard Preparation

About 10 mg of CHL were transferred into a 100 mL, 65 mL of the diluent was added, and the solution was dissolved by sonication for 30 min and made to volume. The final solution was taken for analysis using the given chromatographic conditions.

##### 2.5.3.2 Sample Preparation

About 1 g of the sample was dissolved in 40 mL of the diluent in a beaker, transferred quantitatively into a 100 mL volumetric flask, and then made up to volume using the diluent. The final solution was taken for analysis using the given chromatographic conditions.

## 3 Results and Discussion

### 3.1 Method Development and Optimization

**FIGURE 1:**
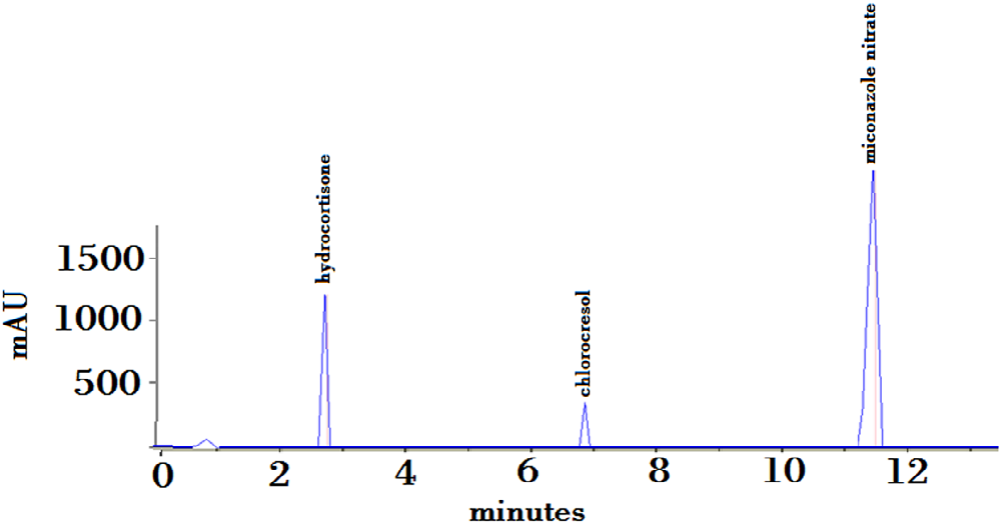
Chromatogram for hydrocortisone, chlorocresol and miconazole nitrate. Conditions: injection volume = 10 µL; column: Zorbax Eclipse plus C18 150 × 4.6 mm^2^, detector wavelength 5 µm; column temperature = 30°C; *λ* = 230 nm; flow rate = 1 mL/min; detector = UV; gradient program: 0 min, 60AAC:40ACN; 4 min, 60AAC:40ACN; 9 min, 5AAC:95ACN; 13 min, 5AAC:95ACN and post‐run time of 3 min.

The results of all optimization steps are summarized in Tables 3 and 4. The maximum UV absorption for MCN, CHL and HCA were 230, 231 and 231 nm, respectively. In this study, 230 nm was selected for use as the detector wavelength. The three molecules are soluble in methanol; therefore, methanol was selected as the solvent of extraction in a water bath to allow the dispersing of the cream. Acetonitrile was selected as the organic modifier for the mobile phase because it is a stronger solvent; hence, it resulted in the early elution of late‐coming peaks. AAC buffer was selected as the buffer at pH 5.0 because this was the pH, which resulted in peaks with the best asymmetry and good resolution. The phosphate buffer resulted in high back pressures. A 150 mm C18 column was selected for the method because the C18 column resulted in the delay of the elution of the first peak. A C8 column resulted in the first peak being eluted before 2 min, but with a C18 column, the peak eluted after 2 min. Gradient elution was selected as the MCN peak took longer to elute using the initial conditions. A flow rate of 1 mL/min was selected to shorten the run time, and a post time of 4 min was selected to equilibrate the system back to the initial conditions. An injection volume of 10 µL was selected for the method because this was optimum for the visibility of the three molecules, Figure 1. Furthermore, it was noted that results at 30°C and 35°C were the same, but 30°C was accepted so as to minimize the consumption of energy at a higher temperature.

**TABLE 3:** A summary of the optimization results for the mobile phase and gradient elution program.

Parameter	Observations	Decision
Mobile phase		
(1) 70:30 acetate buffer pH 7:CAN	HCA retention time 4 min and broad, CHL retention time 7 min, MCN peak unretained up to 20 min	Rejected
(2) 60:40 acetate buffer pH 7:CAN	HCA retention time 2.4 min and broad, CHL retention time 6.4 min, front tailing, MCN peak unretained up to 20 min	Rejected
(3) 60:40 acetate buffer pH 5:CAN	HCA retention time 2.8 min narrow asymmetric peak, CHL retention time 7 min, narrow peak, MCN peak unretained up to 20 min	Considered for initial gradient conditions
(4) 60:40 phosphate buffer pH 5:CAN	HCA retention time 2.8 min, asymmetric peak, CHL retention time 6.8 min, narrow peak, MCN peak unretained up to 20 min (high system back pressure)	Rejected
(5) 70:30 acetonitrile:acetate buffer pH 5	HCA unretained, chlorocresol at 4 min, MCN unretained up to 10 min	Rejected
(7) 60:40 acetate buffer pH 7:MEOH	HCA retention time 3.5 min asymmetric peak, CHL ret time 7 min narrow peak, MCN peak unretained up to 20 min	Rejected
Gradient program A: Buffer B: ACN		
(1) 60:40 (A:B) 0–6 min, 30:70 (A:B) 6.01–20 min	HCA retention time 2.9 min, asymmetric peak, CHL retention time 7.2 min, narrow peak, MCN retention time 12 min with broad peak	Rejected
(2) 60:40 (A:B) 0–6 min, 85:15 (A:B) 6.01–20 min	HCA retention time 2.9 min, asymmetric peak, CHL retention time 7.2 min, narrow peak, MCN retention time 13 min with sharp peak shapes	Rejected
(3) 60:40 (A:B) 0–6 min, 90:10 (A:B) 6.01–20 min	HCA retention time 2.9 min, asymmetric peak, CHL retention time 7.2 min, narrow peak, MCN retention time 12 min with sharp peaks	Accepted

Abbreviations: CHL, chlorocresol; HCA, hydrocortisone; MCN, miconazole nitrate.

**TABLE 4:** A summary of the optimization results for the variation of flow rate, column temperature, injection volume and column type.

Parameter	Observation	Decision
Flow‐rate variation		
(1) 1 mL/min	HCA retention time 2.9 min, asymmetric peak, CHL retention time 7.2 min, narrow peak, MCN retention time 12 min with sharp peaks	Accepted
(2) 1.5 mL/min	HCA retention time 1.7 min, asymmetric peak, CHL retention time 5.8 min, narrow peak and MCN retention time 10 min with sharp peaks	Rejected
Column temperature		
(1) Ambient	HCA retention time 2.9 min, asymmetric peak, CHL retention time 7.2 min, narrow peak and MCN retention time 12 min with sharp peaks	Rejected
(2) 30°C	HCA retention time 2.7 min, asymmetric peak, CHL retention time 6.8 min, narrow peak and MCN retention time 11.3 min with sharp peak shapes	Accepted
(3) 35°C	HCA ret time 2.7 min asymmetric peak, CHL retention time 6.8 min, narrow peak and MCN retention time 11.3 min with sharp peak shapes.	Rejected
Injection volume		
(1) 20 µL	High detector intensity for MCN peak, results in detector saturation	Rejected
(2) 10 µL	Moderate detector intensity for MCN	Accepted
Column variation		
(1) 15 cm C8, 5 µm Zorbax Eclipse Plus ODS	HCA retention time 2.6 min, asymmetric peak, CHL retention time 6.5 min, narrow peak and MCN retention time 11 min with sharp peaks	Rejected
(2) 15 cm C18, 5 µm Zorbax Eclipse Plus ODS	HCA retention time 2.7 min asymmetric peak, CHL retention time 6.8 min, narrow peak and MCN retention time 11.3 min with sharp peaks	Accepted

Abbreviations: CHL, chlorocresol; HCA, hydrocortisone; MCN, miconazole nitrate.

### 3.2 Method Validation

#### 3.2.1 Specificity

The mobile phase was injected into the chromatographic system as well as the placebo. There is no interference, which should be apparent after the visual comparison of standard and sample chromatograms. In this study, no peaks were observed at the retention times for HCA, CHL and MCN. This indicates that the method is specific as it can only detect the molecules of interest as shown in Figure 1.

#### 3.2.2 Precision

The results for precision from the current study are summarized in Table 5. All the three molecules had their RSDs not more than 2% after being injected six times on both Days 1 and 2 (Table 4). The results were accepted against FDA guidelines. Furthermore, the system suitability results were acceptable for the standards of all the three molecules based on FDA guidelines; that is, %RSD was less than 2, and asymmetry was less than 2.0 for all the five injections (Table S1).

**TABLE 5:** RSD for six injections of chlorocresol (CHL), hydrocortisone (HCN) and miconazole nitrate (MCN).

	Day 1			Day 2		
	CHL	HCN	MCN	CHL	HCN	MCN
	Amount (%)	Amount (%)	Amount (%)	Amount (%)	Amount (%)	Amount (%)
Mean	103.87	96.67	102.62	104.44	97.80	102.82
SD	0.56	0.45	0.40	0.99	0.51	0.50
%RSD	0.54	0.47	0.39	0.95	0.52	0.49

#### 3.2.3 Accuracy

The accuracy of the method was determined by spiking a known concentrate of the analyte and the percentage recovery calculated (Table S2). The results indicated that the mean percentage recoveries at 80, 100 and 120 of the intended concentrations are within specifications for the three molecules according to the FDA guidelines.

#### 3.2.4 Linearity

The linearity of the three molecules was determined at five concentrations in the range 80%–120%. The calibration plots for each molecule were all within specifications. The correlation coefficients for HCA, MCN and CHL were 0.9963, 0.9963 and 0.9954, respectively. These results indicate that the linearity for the three molecules is within the specifications for linearity from the FDA guidelines. All the three molecules had a correlation coefficient above 0.995, close to unity.

### 3.3 Solution Stability

This study was conducted to establish the stability of the three molecules using the newly developed method. It was done for a period of 24 h, and the results are given in Table S3. The % differences were 0.4, 0.8 and 0.2 for HCA, CHL and MCN, respectively. The solution stability for the three molecules was well below the acceptable 5% limit.

**TABLE 6:** Variation of results as a result of change of method parameters.

Temperature 28°C				Temperature 32°C		
Sample	MCN	CHL	HC	MCN	CHL	HC
	Amount (%)	Amount (%)	Amount (%)	Amount (%)	Amount (%)	Amount (%)
Mean	102.74	105.88	97.11	102.63	100.28	97.24
Flow rate @ 0.9 mL/min				Flow rate @ 1.1 mL/min		
Sample	MCN	CHL	HC	MCN	CHL	HC
	Amount (%)	Amount (%)	Amount (%)	Amount (%)	Amount (%)	Amount (%)
Mean	102.39	102.73	96.85	102.71	104.77	97.30

Abbreviations: CHL, chlorocresol; HCA, hydrocortisone; MCN, miconazole nitrate.

#### 3.3.1 Robustness

Robustness for this particular study was conducted by changing the column temperature to 30°C ± 2°C as well as changing the flow rate to ±10% of the 1 mL/min. The results are summarized in Table 6. The results for each molecule were all within acceptable specifications, that is, 95%–105% range. These results indicate that the slight changes in the method parameters did not affect the outcome of each chromatographic analysis. Although a change in retention times was observed, the change was uniform for the sample and standard, hence giving acceptable results.

### 3.4 Method Applicability

The validated method was used to analyse a commercial cream. The cream was also analysed using other previously validated methods, and the results from the sets were compared using the Student *t*‐test and *F*‐test to confirm its applicability. It was hypothesized that mean of HCA, CHL and MCN from developed method is not equal to mean of HCA or MCN from USP method. The statistical analysis for HCA has shown that its output, that is, *T* = 1.405 with 10 degrees of freedom and *p* value = Sig.(2‐tailed) = 0.195, which is more than 0.05. The *F*‐test had an output of *F*(1,10) = 1.97 and a *p* value = 0.195, which is greater than 0.05. Therefore, there is no significant difference between the two methods, and the method is accepted for this application. From the MCN output, *T* = −1.533 with 10 degrees of freedom and *p* value = Sig.(2‐tailed) = 0.162, which is more than 0.05. The *F*‐test results gave the output *F*(1,10) = 2.35 with *p* value = 0.162, which is greater than 0.05. Therefore, it is also inferred that the method is comparable to well‐established protocols. From the statistical analysis of CHL data, the output was *T* = −1.718 with 10 degrees of freedom and *p* value = Sig.(2‐tailed) = 0.142, which is more than 0.05. Again, there is no significant difference between the two methods against chromatographic analysis of CHL. The *F*‐test gave an output of *F*(1,10) = 2.95 and *p* value = 0.142, which again is greater than 0.05, suggesting there is no significant difference between the two methods. The results from the recovery studies using the newly validated method are as follows: MCN (102.1%), HCA (97.3%) and CHL (105.9%). In comparison with the results obtained from an already existing USP method MCN (100.9%), HCA (98.5%), and CHL (103.9%), there is no statistically significant difference between the two methods.

### 3.5 Greenness and Sustainability of the Method

The AGREE tool was used to assess the developed method greenness. The method yielded a score of approximately 0.54 (Table 7), suggesting an average environmental friendliness. A score of 0.4–0.59 represents moderate green performance [21, 22]. The method has the following advantages: no derivatization, low sample volume used and intermediate energy usage. However, its overall greenness and sustainability assessment was negatively impacted by the use of acetonitrile as an organic modifier and the generation of solvent waste.

**TABLE 7:** Principles covered by AGREE tool along with scoring.

Principle	Description	Score
1. Sample preparation	Moderate complexity	0.5
2. Sample size	Small sample size 10 µL	0.9
3. In situ instrument	Lab‐based HPLC used (not in situ)	0.2
4. Number of steps	Extraction + filtration + injection	0.5
5. Automation and miniaturization	HPLC not miniaturized but is automated	0.6
6. Derivatization	No derivatization	1.0
7. Waste generation	Acetonitrile‐based gradient thus moderate to high solvent waste	0.3
8. Throughput (analysis time)	≈13 + 3 min post run = 16 min	0.5
9. Energy consumption	30°C (low) but HPLC running automatically	0.6
10. Reagent toxicity	Acetonitrile is hazardous and toxic	0.2
11. Safety of reagent	Organic solvent risk	0.3
12. Waste treatment	Standard disposal	0.5

Abbreviation: HPLC high‐performance liquid chromatography.

The developed method demonstrated various environmentally favourable characteristics, such as small injection volume (10 µL), no derivatization steps, a moderate column temperature (30°C) and automated operation. All these contributed to less energy consumption and sample handling. The method's sustainability was thus intermediate.

## 4 Conclusion

On the basis of the findings of this work, the developed method is selective, precise, accurate and robust and has a good working linear range. It is less time consuming and economic as compared to individual analysis of the three molecules in the cream. The newly developed and validated method can be used for the simultaneous determination of MCN, CHL and HCA in a pharmaceutical dosage form.

## Author Contributions

**Netai Mukaratirwa‐Muchanyereyi**: conceptualization, methodology, software, data curation, investigation, validation, formal analysis, supervision, visualization, project administration, resources, writing – original draft, writing – review and editing. **Pretty Chihiya**: conceptualization, methodology, software, data curation, investigation, validation, formal analysis, visualization, resources, writing – review and editing. **Stephen Nyoni**: methodology, data curation, investigation, validation, formal analysis, visualization, writing – original draft, writing – review and editing.

## Conflicts of Interest

The authors declare no conflicts of interest.

## Supporting information




**Supporting File**: ansa70085‐sup‐0001‐SuppMat.docx

## Data Availability

The data that support the findings of this study are available from the corresponding author upon reasonable request.
