# Supplementary material for: Method Development and Validation for the Simultaneous Analysis of Miconazole Nitrate, Hydrocortisone and Chlorocresol in a Pharmaceutical Topical Cream by Reverse Phase Liquid Chromatography
Source: Anal Sci Adv. 2026 Apr 27;7(1):e70085. doi: 10.1002/ansa.70085 (PMC13112079; doi:10.1002/ansa.70085)
Supplement: Supplementary file 1 — Supporting File: ansa70085‐sup‐0001‐SuppMat.docx [file ANSA-7-e70085-s001.docx]

## Supplementary Information (SI)

**SITable 1: System suitability results for five injections of HCA, CHL and MCN**

|  | **HCA area/ x10^6^** | **CHL area/ x10^6^** | **MCN area/ x10^6^** |
| --- | --- | --- | --- |
| **Mean** | 507.4 (1.15*) | 87.4(1.13*) | 1191.6 (1.21*) |
| **SD** | 0.74 | 0.18 | 1.48 |
| **% RSD** | 0.15 | 0.21 | 0.12 |

* Values in brackets indicate average peak asymmetry.

## SI Table 2. Mean % Recoveries of HCA, MCN, and MCN

| Concentration | Mean Area (n=3) | Mean Conc. Recovery (n=3) | Mean % Recovery |
| --- | --- | --- | --- |
| HCA |  |  |  |
| 200µg/ml | 39473454.7 | 194.5±2.40 | 97.2±1.20 |
| 250µg/ml | 502612315 | 247.6±1.15 | 99.1±0.46 |
| 300µg/ml | 600814085.7 | 296±0.45 | 98.7±0.15 |
| MCN |  |  |  |
| 400µg/ml | 952083792.7 | 399.49±3.30 | 99.9±0.83 |
| 500µg/ml | 1192271648 | 500.26±2.04 | 100.1 ±0.41 |
| 600µg/ml | 1387062401 | 582±3.22 | 97±0.54 |
| CHL |  |  |  |
| 20µg/ml | 65795822.67 | 18.82± 0.07 | 94.1± 0.35 |
| 25µg/ml | 84547639 | 24.18±0.01 | 96.7±0.50 |
| 30µg/ml | 103552468 | 29.62±0.14 | 98.7±0.49 |

## SI Table 3: Solution stability results

| **Identity** | **Initial results** | | | **Results after 24 hours** | | |
| --- | --- | --- | --- | --- | --- | --- |
| **Sample #** | **% HCA** | **% CHL** | **% MCN** | **% HCA** | **% CHL** | **% MCN** |
| 1 | 96.49 | 103.39 | 102.29 | 95.91 | 102.58 | 102.10 |
| 2 | 96.51 | 103.67 | 102.47 | 96.33 | 102.74 | 102.48 |
| **Mean** | 96.5 | 103.5 | 102.3 | 96.33 | 102.74 | 102.48 |
| **% Difference** |  |  |  | 0.4 | 0.8 | 0.2 |
